# The influence of evaluative right/wrong feedback on phonological and semantic processes in word learning

**DOI:** 10.1098/rsos.171496

**Published:** 2018-09-05

**Authors:** Saloni Krishnan, Elise Sellars, Helena Wood, Dorothy V. M. Bishop, Kate E. Watkins

**Affiliations:** Department of Experimental Psychology, University of Oxford, Anna Watts Building, Radcliffe Observatory Quarter, Oxford OX2 6GG, UK

**Keywords:** word learning, foreign language learning, vocabulary, phonological, semantic, feedback

## Abstract

Feedback is typically incorporated in word learning paradigms, in both research studies and commercial language learning apps. While the common-sense view is that feedback is helpful during learning, relatively little empirical evidence exists about the role of feedback in spoken vocabulary learning. Some work has suggested that long-term word learning is not enhanced by the presence of feedback, and that words are best learned implicitly. It is also plausible that feedback might have differential effects when learners focus on learning semantic facts, or when they focus on learning a new phonological sequence of sounds. In this study, we assess how providing evaluative (right/wrong) feedback on a spoken response influences two different components of vocabulary learning, the learning of a new phonological form, and the learning of a semantic property of the phonological form. We find that receiving evaluative feedback improves retention of phonological forms, but not of semantic facts.

## 1. Introduction

Learning new words is something humans do constantly. Even in adulthood, we acquire new words when travelling to foreign countries (for instance, ‘salida’ for exit), learning new skills (for instance, ‘quenelle’ when baking), or even when new trends emerge (for example, ‘hygge’). Indeed, vocabulary knowledge is a crucial predictor of school success (reviewed in [1]). Adding words to our vocabulary allows us to talk about newly acquired concepts and ideas with ease. Although this process seems effortless, for foreign language learners or for those struggling with language learning [2,3], word learning can be a slow and painstaking process. A question that is consequently relevant in both educational [1] and clinical settings [4] is how we might be able to optimize word learning. In paradigms that involve explicit training, feedback is thought to play a key role, providing an error signal for learners to correct or refine their performance. In this study, we investigated how feedback about the correctness of a response would affect spoken vocabulary learning, an issue of theoretical and practical interest to both neurobiologists [5] and cognitive psychologists [6]. Spoken vocabulary learning is thought to involve at least two kinds of learning, learning a novel phonological form and learning semantic information about a target [7]. We assessed whether using feedback to direct attention to the phonological form of a word (learning a new sequence of sounds like ‘perklisterent’), or on its semantic attributes (a fish that swims around in circles), could differentially improve learning of these specific aspects of vocabulary, and to what extent this generalized to the other aspect of word learning.

At first glance, the value of feedback appears obvious, and as such, it is used without question in learning paradigms and applications. Feedback is incorporated in training packages for foreign vocabulary and language learning, such as DuoLingo and Rosetta Stone, as well as typical language intervention packages. Feedback is thought to counteract some of the disadvantages introduced by retrieval-based approaches, which introduce tests at various points of the learning cycle [8]. Although such testing is thought to be a powerful way to enhance later recall [9], participants might remember their own error responses instead of the target. Indeed, in patients with memory impairments [10,11], as well as healthy controls [12–14], an advantage for errorless over error-prone learning has been observed in various domains of learning. The error signal provided by feedback is thought to mitigate the risk that errors produced during the learning process will be reinforced. Furthermore, feedback can serve as a means of directing attention to relevant information, perhaps changing the way learners monitor features of the environment. In studies involving learning of facts or definitions, feedback following testing has been shown to lead to substantially improved memory for the items in question [15–17].

Despite the demonstrated value of feedback for learning semantic facts and knowledge, the value of feedback for vocabulary learning processes has been called into question. It is important to note that the term ‘feedback’ is used quite variably within this literature, for example, to refer to conditions where participants are made aware if they have performed correctly or incorrectly [4,18], those where the correct answer is provided [19,20] and those where more explanatory feedback on performance is given [20,21]. Here, we define evaluative feedback as a cue that highlights the accuracy of a response, without correcting it, but with an opportunity to learn the correct response later. We contrast this with a no-feedback condition, where there is no cue highlighting response accuracy, but learners do receive a later opportunity to learn the correct response. Some researchers have argued that such evaluative feedback might be unnecessary for learning associations between pseudowords and pictures [4], and that implicitly learning the frequency of co-occurrences between pairs was sufficient. The authors argue that evaluative feedback makes little difference in language learning, and support this argument by highlighting the implicit nature of children's lexical and phonological learning [22,23]. In another study involving learning associations between pairs of words (a native language word and one from a foreign language), the authors argued that the value of feedback is limited to when the correct answer was provided as feedback, and that simply providing information about whether the response was right or wrong actually worsened learning [17]. However, their design made it difficult to disassociate the value of the additional exposure to the target word from the evaluative signal provided by feedback. Put differently, giving participants an additional opportunity to encode the target might enhance learning regardless of feedback after performance. In addition, existing studies of word learning and feedback have tended to provide feedback only once at the end of learning [6,15,16,19]. If evaluative yes/no feedback is provided at the end of learning, with an unconstrained set of responses, and no further opportunity to learn, it is easy to understand why participants would not derive any further benefit. By contrast, providing a later review of materials after prior evaluative feedback might be more effective, because it would allow the participant to revise low-confidence responses [18].

It is plausible that evaluative feedback might have different impacts on phonological and semantic learning. In previous word learning studies, semantic and phonological processes appear to show different profiles of learning and retention, suggesting that learning of these kinds of information does not follow the same trajectory [24,25]. Phonological knowledge is also considered more implicit in comparison to semantic knowledge, which could mean that providing explicit feedback would give less benefit. To our knowledge, no studies involving feedback have contrasted these processes, and current studies on the influence of feedback on either of these domains do not lead to clear predictions. In a study that involved learning phonological contrasts (such as the difference between a /r/ and a /l/ sound, contrasts that are unfamiliar to Japanese monolinguals), evaluative feedback was beneficial [26,27]. However, evaluative feedback was argued to be worse than no feedback at all when it came to learning semantic associations [19]. Although this may at first glance look like a semantic/phonological divide, differences in task design, context and prior knowledge are likely to influence the benefit received from feedback. In constrained environments with few choices, such as learning phonological contrasts, or environments where listeners possess general semantic knowledge, learners may be able to better hone their predictions and benefit from evaluative feedback. In contrast, this process may be much harder when learning a novel sequence of sounds, such as pseudowords, as listeners will be less explicitly aware of constraints. The two domains are also likely to be characterized by different kinds of errors; for example, errors in the phonological domain might be characterized by an absence of a response, whereas those in the semantic domain might involve a closely related response. Therefore, feedback might be more likely to enhance recall of related answers, and eventually the correct answer in the semantic domain [15,28]. On the other hand, the greater difficulty of learning may lead to an enhanced effect of feedback in the phonological condition, as participants might be more likely to come up with strategies to remember the material [28].

Furthermore, another argument about the value of feedback for vocabulary learning is that although immediate feedback might lead to greater learning efficiency in the short term (typically immediately after training), it is unclear whether this benefit is sustained over longer durations, for instance, a week after learning [19,29]. To address these questions about the role of feedback in vocabulary learning, and whether it has a distinct effect on phonological and semantic learning processes, we focused on whether evaluative feedback plays a role in word learning over a relatively long retention period (one week).

It may be impossible to assess the effect of feedback on phonological and semantic learning separately, because the very presence of semantic context is known to influence the integration and consolidation of new phonological forms. Phonological representations formed in rich semantic contexts are more robust over the long term [30,31]. A theoretical account of this effect posits that the phonological forms become embedded in a semantic net, and these rich semantic associations may make these words easier to retrieve at later time points [32]. However, this effect of context has only been observed in paradigms that contrast sparse word learning paradigms to semantically rich ones [30,31,33]. Therefore, an issue that these studies are unable to assess is whether the presence of semantics simply enhances overall attention, which leads to better learning. In our study, participants are exposed to the same stimuli, but only asked to respond to one aspect of each item (either the phonological form, or the semantic association). This gives us a novel and better controlled means of assessing whether focusing on learning phonological forms or semantic features has a differential effect on lexical retention. If the semantic integration account is correct, we would predict better learning of phonological forms after training on repeating the semantic information, rather than repeating the phonological information itself. Although this seems counterintuitive, this would support the view that providing semantic information during word learning helps form lasting lexical representations [30]. However, if the differences in sparse versus semantically rich tasks are driven by attention or motivation, we would expect to see no difference in learning of word forms between the phonological and semantic versions of the task, or we might see slightly enhanced learning in the phonological context.

In this study, we used a training regime that involved mapping novel phonological forms embedded in meaningful sentences to novel visual referents. We varied the type of feedback participants received during learning (no feedback/evaluative feedback) as well as the focus of their learning (semantic/phonological). The learning phase of the study involved repetition and cued recall of either semantic attributes or the phonological form of the target. Therefore, half the participants (*N* = 40) received evaluative feedback on their retention of the semantic information about the referent, while being incidentally exposed to the phonological form, and the other half (*N* = 40) received evaluative feedback on their production of the phonological form, while being incidentally exposed to the semantic feature. Word learning was assessed a week after learning using cued recall of phonology and semantics. We tested the following hypotheses:

(A) evaluative feedback leads to greater word learning relative to no feedback;
(B) the training condition (semantic/phonological) leads to better performance on related cued-recall tests (semantic/phonological, respectively); and
(C) feedback on semantic attributes enhances cued recall of semantics to a greater extent than feedback on phonological form, and vice versa.

## 2. Methods

### 2.1. Pre-registration, data and material release

This project was pre-registered on the Open Science Framework; the registration can be viewed at https://osf.io/xdevy/. All anonymized data collected as part of this study are openly available on the Open Science Framework (OSF), on https://osf.io/bdfqy/ [34]. The code to run the training and assessment programs is also available on the OSF at the same link. We have also uploaded materials, such as the images used and pseudowords, so that interested readers can reproduce the training and assessment programs. However, we note that as the images are commercial stimuli, their use outside of the context of this training program is prohibited (i.e. readers cannot use these to design a new training study).

### 2.2. Participants

We recruited 80 participants (40 each in the semantic and phonological training conditions; see §2.11 for further details). Our inclusionary criteria were healthy native English-speaking adults aged between 18 and 40 years with normal or corrected-to-normal vision. Exclusionary criteria were any known neurological disorder, speech, language or hearing disorders, and colour blindness. Participants were recruited through the university's research participation pool, and through poster and email advertisements on departmental mailing lists. Participants had to complete two sessions spaced exactly one week apart. Two participants did not complete the second session; we consequently replaced their data with two new participants who did complete both sessions. The experiment took place on university premises, in a quiet testing room free from distractions. In order to provide participant-specific feedback, researchers tested participants individually.

### 2.3. Experimental design

**Figure 1.**
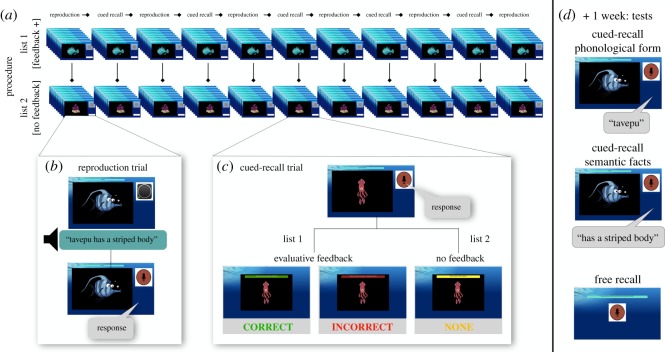
Schematic of experimental design. Participants are randomly assigned to either the phonological or semantic condition. (*a*) During training, participants complete 11 alternating blocks of reproduction and cued recall. In each block, they encounter 12 items from List 1, and 12 items from List 2. Items in one of the lists are designated as those receiving feedback. (*b*) In a reproduction trial, participants hear a fact, like ‘Tavepu has a striped body’. They then have to reproduce either the phonological form (Tavepu) or the fact (has a striped body). No feedback is given. (*c*) In a cued-recall trial, participants encounter a visual target, and are cued to give their response (the phonological form or the semantic fact, based on the training condition). An experimenter marks each spoken response as correct/incorrect. However, only items in one of the lists receives evaluative feedback (here, items in List 1); for items in the other list, participants see a screen acknowledging their response. (*d*) Tests are completed one week after training. All 24 items are tested using cued-recall tests of phonology and semantics. Here, the first set tests knowledge of the phonological forms associated with each referent and the second set of tests assesses memory for the semantic facts associated with each target. Participants also complete a free-recall test. No feedback is provided during tests.

The training and testing tasks were implemented using custom Matlab software. During the course of the training, participants attempted to learn 24 novel and orally presented pseudowords. Each pseudoword was paired with a visual target and embedded in a sentence describing a semantic feature about the target (for example, ‘Tavepu has a striped body’). Participants were assigned to one of two training conditions, phonological or semantic, and they were expected to overtly produce either the pseudoword or the semantic feature associated with the target. They received evaluative (correct/incorrect) feedback for 12 pseudowords during recall blocks, and no feedback for the remaining 12 pseudowords. A week following training, participants completed three recall tasks to determine how much information they had retained about the targets they previously encountered (cued recall of phonology, cued recall of semantics, free recall). This experimental design is illustrated in figure 1.

### 2.4. Stimuli

Visual stimuli were chosen from a commercially available image database (shutterstock.com). We picked 12 pictures of underwater creatures and 12 pictures of underwater plants. The pictures were chosen to be easily distinguishable and to belong to separate categories. A further consideration was that they should not be associated with familiar verbal labels (e.g. a goldfish).

Two pseudoword lists, comprising 12 words each, were used in this study. Each list consisted of six two-syllable words, and six three-syllable words. The two lists were matched with respect to number of syllables, stress pattern and consonant clusters. They were derived using the program ‘Wuggy’ [35], which allows for the generation of written polysyllabic pseudowords that follow the phonotactic constraints of English. When Wuggy is given a word in English, it extracts the sub-syllabic structure of this template word, and generates pseudowords that match the template in sub-syllabic structure and transition frequencies. Using output generated by Wuggy, we also ensured that the two pseudoword lists were matched for neighbourhood size and density, as well as the number of orthographic neighbours at edit distance one (words possible by substituting, inserting or deleting a single letter).

Pseudowords were then arbitrarily matched to visual stimuli. A set of statements about the visual stimuli was then constructed and divided into two lists. Each list comprised statements about plants and creatures, with six observable facts, (for example, ‘Tavepu has a striped body’) and six abstract ones (for example, ‘Ivogun grows in shallow waters'). The observable facts corresponded to specific visual features of the stimuli but the abstract ones were arbitrary. The lists were balanced for the number of semantic features, syntactic complexity and length.

Pilot testing was conducted with eight participants (four assigned to each condition) who were not enrolled in the final study, who received between five and six blocks of reproduction practice (see electronic supplementary material, appendix S3, for further details). Five or six cued-recall blocks were interleaved in between these blocks. Accuracy was calculated by averaging the number of correct responses in the cued-recall blocks. The chosen phonological and semantic stimuli showed accuracy rates of 79.17% and 87.5% in the final recall block (for the phonological and semantic training condition, respectively).

Using the sets of visual referents, pseudowords and fact lists described above, we created two stimuli sets (Lists 1 and 2; see electronic supplementary material, appendix S1), where we counterbalanced the number of syllables (two/three) and type of fact (observable/abstract). Optimizing the counterbalancing of the type of picture (underwater plant/creature) was less important given that it was not the focus of study.

### 2.5. Positive controls

In studies that have explored phonological learning, a consistent effect relates to the length of the words to be learned. This effect shows that longer words (or words with more syllables) are harder to learn relative to shorter words [36]. In terms of semantic learning, a similar effect is the level of abstractness. Observable features are found to be easier to learn than abstract ones [37]. By including the factors of word length and observability in our design, we had an outcome-neutral way to determine that any word learning data follow expected patterns with respect to both phonological and semantic learning. Specifically, we predicted that the three-syllable words and the abstract facts would be recalled less accurately at test than the two-syllable words and the observable facts, respectively.

### 2.6. Testing schedule

Training occurred during the first session, which was approximately 45 min long. Participants provided demographic details and then completed the training phase, which involved playing the word learning game. They did not complete any tests in the first session. Participants completed the second testing session exactly one week after the training session. The testing session occurred at approximately the same time of day as training for each participant. In the second session, participants were given the two cued-recall and free-recall tests. They were then compensated for their time. The second session lasted for approximately 15 min.

### 2.7. Procedure for training phase

Participants were expected to learn the names of 24 underwater living things during the training phase, and semantic features associated with each of these. To learn this information, they completed 11 blocks of training, during which they repeated or retrieved the target information. In each block, participants were exposed to all 24 targets, blocked by list (12 underwater living things in each list). Picture order within each list was randomized, so participants did not learn the targets in any specific order. The order in which each list block appeared was counterbalanced across participants. Half the participants encountered words in List 1 followed by those in List 2 in each block, and the other half encountered List 2 before List 1. The list that received feedback was also counterbalanced across participants, with half the participants receiving feedback for words in List 1 but not List 2, and the other half receiving feedback for List 2 but not List 1. Consequently, there were four arms to the counterbalancing: (1) List 1 with feedback followed by List 2 without; (2) List 1 without feedback followed by List 2 with feedback; (3) List 2 with feedback followed by List 1 without; and (4) List 2 without feedback followed by List 1 with feedback. Ten participants were assigned to each arm, so these conditions were counterbalanced in the study.

At the start of each block of the training trials, participants were given instructions specific to the condition. Blocks 1, 3, 5, 7, 9 and 11 were the *Reproduce* blocks. In these blocks, participants were asked to overtly repeat the pseudoword or semantic feature, according to the training condition. In each trial, participants were shown the target visual referent. A speaker icon was shown, and participants heard a sentence comprising the pseudoword and a semantic feature about the referent. The participants were then given a visual cue (a microphone icon turning red) to speak. Based on their training condition, participants were expected to repeat either the pseudoword or the semantic feature. Participants were instructed to repeat the pseudoword or semantic feature exactly as spoken. They had 3 s to respond from the time the microphone turned red. They did not receive any feedback on their production, but simply moved on to the next trial, where they encountered the next visual referent. They also had an opportunity to take a short break at the end of each block. Blocks 2, 4, 6, 8 and 10 were the cued *Recall* blocks. At the start of the block, participants were told to overtly recall from memory the pseudoword/semantic feature associated with the visual target presented. In each trial, they were shown a picture on screen (they did not hear anything). The microphone icon turned red, and they had three seconds to say the pseudoword/semantic feature. For both pseudowords and semantic features, only the exact sequence of sounds or verbatim wording was acceptable. A trained researcher coded their response as correct/incorrect in real time. Based on the feedback condition for the stimulus, participants either saw a screen acknowledging their response (no evaluative feedback), or a screen that gave them information about whether their response was correct or incorrect (evaluative feedback).

### 2.8. Recall tests

Regardless of which training condition participants were assigned to, they completed three recall measures: cued recall of pseudowords, cued recall of semantic features and free recall of each list of words they encountered. In all cases, the cue was the visual stimulus seen in training a week earlier. The order in which cued recall of phonological forms and cued recall of semantic features was tested was counterbalanced. Free recall was always measured after testing cued recall.

To assess cued recall of phonology, participants were instructed to try and provide the pseudoword associated with the picture cue shown. The cue was the visual referent; they were not provided with the first phoneme or other semantic details. They were encouraged to guess if they were not completely sure of the answer, and say ‘pass’ only if they absolutely could not recall the target. They completed 24 trials, comprising targets from both Lists 1 and 2. The order in which Lists 1 and 2 appeared was counterbalanced, and the order of specific items in the list randomized. In each trial, the visual referent associated with the target was shown on the left side of the screen. When they received an indication to speak (the microphone turning red), participants were expected to produce the pseudoword. They had 3 s to articulate their response before moving on to the next trial. Responses were audio-recorded; participants received no feedback on their production. The procedure to assess cued recall of semantic features was identical to that used to assess cued recall of phonology, but rather than recall the pseudoword, participants were instructed to recall the semantic feature associated with the visual referent. In the free-recall tests, participants were instructed to recall the names of all the living things they had encountered during training. They had one minute to respond and their responses were audio-recorded. If participants did not provide any responses for 30 s, they received a prompt from the experimenter, ‘Can you think of any underwater plants/animals you encountered?’. Participants did not receive feedback on their responses during tests in the second session.

### 2.9. Data coding and reliability

We scored all audio-recorded productions during the cued-recall phase as accurate (1) or inaccurate (0). A second rater coded all the words and statements produced in the final cued-recall tests; inter-rater reliability (*r* = 0.97, *p* < 0.001) was higher than the minimum threshold we set in our Stage 1 submission (*r* = 0.85).

Accuracy scores were averaged to calculate recall accuracy according to the different training conditions (semantic/phonological), recall test (semantic/phonological) and feedback (evaluative/none).

### 2.10. Outlier detection

We visually inspected data for the presence of outliers. We previously decided to only exclude data identified as outliers using the Hoaglin & Iglewicz [38] outlier labelling rule with *k* = 2.2. Instead of identifying outliers on the basis of standard deviations from the mean, this rule involves multiplying the interquartile range by *k*, and excluding data only beyond this range. To identify outliers without excluding good/poor learners, we calculated the difference between the two feedback conditions in accuracy scores at test. We also calculated the difference in accuracy scores at test between the two recall conditions. We found that none of these values would be classified as outliers using the Hoaglin and Iglewicz rule and consequently did not exclude any data.

### 2.11. Analyses

The specific contrasts we registered to assess support for our hypotheses are detailed below, and our power analysis is available in electronic supplementary material, appendix S2.

#### 2.11.1. Positive controls

Before turning to our experimental hypotheses, our plan specified assessing whether the data showed expected effects of learning, which would indicate that the training had worked as expected.

*Phonological*. Using a 2 × 2 mixed ANOVA, we assessed if there was an effect of word length (two/three syllables) on the cued recall of phonology, also modelling training condition (semantic/phonological). We expected to see a main effect of word length (greater accuracy for two- versus three-syllable words), moderated by an interaction with training condition.

*Semantic*. Using a 2 × 2 mixed ANOVA, we assessed the effect of observability on semantic learning (observable/abstract) on the cued recall of semantics, also modelling training condition (semantic/phonological). We expected a main effect of observability (observable attributes more accurately recalled than abstract ones), moderated by an interaction with training condition.

#### 2.11.2. Hypothesis A: evaluative feedback leads to greater word learning relative to no feedback

Our first hypothesis was quite broad, to help us to establish if feedback had any effect on word learning. We used a 2 × 2 × 2 mixed ANOVA of cued-recall accuracy, modelling the between-subject factor training condition (semantic/phonological) and the within-subject factors cued-recall condition (semantic/phonological), and feedback (evaluative/none). In the presence of a significant main effect of feedback, we planned to confirm that the pattern of means was consistent with our prediction that evaluative feedback would lead to greater word learning relative to no feedback.

#### 2.11.3. Hypothesis B: The training condition (semantic/phonological) leads to better performance on related cued-recall tests (semantic/phonological, respectively)

Our second hypothesis was that we would see some specificity of learning based on the focus of training. To test this, we used the model of cued-recall accuracy described in §2.11.2. Our prediction was an interaction between training condition and cued-recall condition, in that training in the semantic condition would lead to better performance in the semantic cued-recall test, whereas training in the phonological condition would lead to better performance in the phonological cued-recall test. If the interaction between training condition and cued-recall condition was significant, we planned to conduct post hoc independent sample *t*-tests (*p* < 0.05) to assess whether cued recall of semantics was greater when participants receive semantic rather than phonological training, and that participants who received phonological rather than semantic training showed higher cued recall of phonology scores. However, if attention to semantic context improved phonological learning, we expected to find a main effect of training condition.

#### 2.11.4. Hypothesis C: feedback on semantic attributes enhances cued recall of semantics to a greater extent than feedback on phonological form, and vice versa

Our third hypothesis was that feedback has a differential effect on semantic and phonological learning. We used the model described in §2.11.2, with cued-recall accuracy as the dependent variable. Our prediction was that there would be an interaction between the between-subject factor of training condition (semantic/phonological) and the within-subject factor of feedback (evaluative/none), with evaluative feedback given to phonological forms leading to better cued recall of phonological forms, and evaluative feedback given to semantic features leading to better cued recall of semantic features. However, it was also possible that feedback would only have an effect on phonological learning, or that feedback would only have an effect on semantic learning. Other alternatives we considered were feedback having a beneficial effect in one training condition, and a detrimental effect in the other condition. Consequently, if the three-way interaction between training focus, cued-recall condition and feedback was significant, we planned to conduct two 2 × 2 repeated measures ANOVAs to examine the interaction between cued-recall condition and feedback per training focus condition. If the interaction was significant, under a Bonferroni-corrected alpha level of *p* < 0.025, we planned to conduct Bonferroni-corrected paired samples *t*-tests to compare the feedback and the no-feedback condition for each of the cued-recall measures.

Current research suggests that feedback may be more beneficial when participants make errors [15]. If error rates differ substantially between conditions, conclusions drawn about phonological and semantic learning might actually be driven by a difference in learning trajectories during the training phase. We expected that participants would find learning of the phonological forms more challenging than learning of semantic facts. If we observed an interaction between feedback and training condition, we planned to assess if this was driven by difficulty learning the information, or the domain of language processing. To do so, we planned to use a *t*-test to compare error rates for participants in the semantic and phonological conditions (by averaging the number of errors committed in the cued-recall blocks of the training condition for each condition regardless of feedback). If these were not significantly different (*p* > 0.05), we expected any interactions between feedback and training condition to be driven by the language domain rather than processing difficulty. If these were significantly different, we expected that error rates would be similar in the easier phonological condition and the harder semantic condition. We proposed to use these levels to conduct a further ANOVA on whether feedback has distinct effects on learning the two-syllable forms in the phonological condition and learning abstract facts alone. If the results were in agreement with the first dataset, we would conclude that the error rate is unlikely to moderate the predicted interaction. However, if error rates across these conditions were not matched, or the interaction shows a different outcome to the one we predicted, we would interpret our results in light of the difference in error rates.

## 3. Results

### 3.1. Positive controls

Data analysis confirmed that the training was successful as the expected effects of learning were significant.

#### 3.1.1. Phonological

As predicted, we observed a significant main effect of word length in cued recall of phonology, *F*_1,78_ = 50.2, *p* < 0.001, *η*_G_^2^ = 0.07. As predicted, we also detected a significant interaction of word length and training condition, *F*_1,78_ = 26.0, *p* < 0.001, *η*_G_^2^ = 0.04. In the phonological training condition, participants recalled significantly more two-syllable words (M = 4.7, s.d. = 3.7) than three-syllable words (M = 2.5, s.d. = 2.6), *t*_39_ = 7.0, *p* < 0.001, *d* = 1.11. In the semantic training condition, the difference between two-syllable (M = 0.6, s.d. = 1.0) and three-syllable words (M = 0.2, s.d. = 0.7) was smaller and not significant, *t*_39_ = 2.0, *p* = 0.05, *d* = 0.32. It should be noted, however, that the lack of a word length effect in the semantic training condition could be attributed to a floor effect, with very little learning of phonological information in this condition.

#### 3.1.2. Semantic

For cued recall of semantics, we found the expected main effect of observability, *F*_1,78_ = 96.1, *p* < 0.001, *η*_G_^2^ = 0.23, modulated significantly by training condition, *F*_1,78_ = 24.3, *p* < 0.001, *η*_G_^2^ = 0.07. In the semantic training condition, observable facts (M = 9.6, s.d. = 1.7) were recalled with higher accuracy than abstract facts (M = 6.1, s.d. = 3.0), *t*_39_ = 9.0, *p* < 0.001, *d* = 1.43. In the phonological training condition, this pattern of observable facts (M = 1.8, s.d. = 2.1) being recalled more accurately than abstract ones (M = 0.7, s.d. = 1.2) was also a significant difference, *t*_39_ = 4.2, *p* < 0.001, *d* = 0.67, but the difference was smaller and as before there is potential here for a floor effect.

### 3.2. Hypothesis A: evaluative feedback leads to greater word learning relative to no feedback

We did not observe a main effect of feedback, *F*_1,78_ = 0.003, *p* = 0.957, *η*_G_^2^ < 0.0001 on cued-recall performance. Across the two training and cued-recall conditions, performance when evaluative feedback (M = 3.3, s.d. = 3.6) was provided was indistinguishable from when no feedback (M = 3.3, s.d. = 3.6) was provided. However, the interaction between feedback and cued-recall condition was significant. This interaction is described and explored further in §3.4.

### 3.3. Hypothesis B: The training condition (semantic/phonological) leads to better performance on related cued-recall tests (semantic/phonological, respectively)

**Figure 2.**
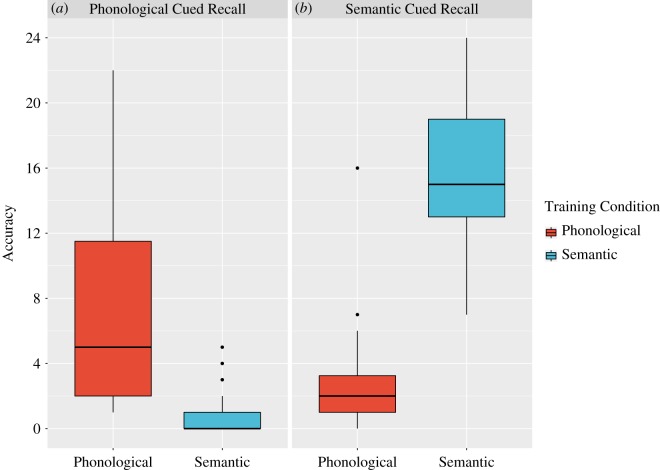
Cued-recall performance in the final test conducted a week after learning, separated by training condition and cued-recall condition. The figure shows accuracy in the (*a*) phonological and the (*b*) semantic cued-recall conditions. For each cued-recall condition, performance is grouped by training condition, with performance following phonological training depicted in red and that of participants who received semantic training shown in blue. Box plots show the median in black, the edges of the boxes represent the interquartile range and the whiskers denote the upper and lower extremes of the distribution (25th or 75th percentile ±1.5 of the interquartile range). Black dots indicate outliers beyond these extremes.

The predicted interaction between training condition and cued-recall condition for accuracy in cued-recall tests was significant, *F*_1,78_ = 279.2, *p* < 0.001, *η*_G_^2^ = 0.57 (figure 2). For cued recall of phonology, those in the phonological training condition (M = 3.6, s.d. = 3.0) performed more accurately than those in the semantic training condition (M = 0.4, s.d. = 0.7), *t*_42.58_ = 6.6, *p* < 0.001, *d* = −1.47; again, note the floor effect for cued recall of phonology following semantic training. Similarly, for semantic cued recall, those in the semantic training condition (M = 7.8, s.d. = 2.2) performed more accurately than those in the phonological training condition (M = 1.2, s.d. = 1.5), *t*_69.6_ = 15.9, *p* < 0.001, *d* = 3.0 (figure 2).

There was also a significant main effect of training condition, *F*_1,78_ = 23.4, *p* < 0.001, *η*_G_^2^ = 0.13, which was due to higher scores on cued recall following semantic training (M = 4.1, s.d. = 4.1), compared with phonological training (M = 2.4, s.d. = 2.8). This indicates that the semantic facts were easier to learn and retain than the novel phonological forms.

### 3.4. Hypothesis C: feedback on semantic attributes enhances cued recall of semantics to a greater extent than feedback on phonological form, and vice versa

We originally hypothesized that, in the phonological training condition, we would find an effect of feedback on cued recall of phonology; and in the semantic training condition, we would see an effect of feedback on cued recall of semantics. An alternative possibility was that one of these effects would be present but not the other, or that feedback would have different effects in both training conditions. This should have been picked up by a three-way interaction between recall, feedback and training condition; or in a two-way interaction between feedback and training condition. The interaction between training condition and feedback that we predicted was not significant, *F*_1,78_ = 3.7, *p* = 0.06, *η*_G_^2^ = 0.003, and neither was the three-way interaction between recall, training condition and feedback, *F*_1,78_ = 1.4, *p* = 0.24, *η*_G_^2^ = 0.001. However, we did observe specificity in the advantage conferred by feedback, as we found a significant interaction between feedback and recall condition, *F*_1,78_ = 5.9, *p* = 0.02, *η*_G_^2^ = 0.005. Feedback improved cued recall of phonology in both semantic and phonological training conditions compared with no feedback, whereas cued recall of semantics in either training condition was not influenced by feedback. Specifically, for phonological recall, items learned across training conditions with evaluative feedback (M = 2.2, s.d. = 3.0) were recalled more accurately than those learned without feedback (M = 1.8, s.d. = 2.6), *t*_79_ = 2.2, *p* = 0.03, *d* = 0.25. In contrast, for semantic recall, items learned in both training conditions with evaluative feedback (M = 4.4, s.d. = 3.8) were recalled as accurately as those learned without feedback (M = 4.7, s.d. = 4.0), *t*_79_ = 1.6, *p* = 0.121, *d* = 0.18. This difference in the effect of feedback across the two types of recall is somewhat surprising, as those in the semantic training condition received no feedback on their phonological recall, and those in the phonological training condition received no feedback on their semantic recall. Therefore, despite the lack of a three-way interaction, we follow up this recall × feedback condition interaction in exploratory analyses below by assessing responses during each training condition (see §3.5.1).

### 3.5. Exploratory analyses

In this section, we report the results of analyses that we had not planned to conduct in advance of collecting the data. Instead, the intention of these analyses is to understand patterns in the data we collected, and generate hypotheses worth testing in the future. It is worth noting that inferential statistics, particularly *p*-values, are difficult to interpret for such exploratory analyses [39]. Consequently, they should be interpreted with caution.

#### 3.5.1. Feedback in the two training conditions

**Figure 3.**
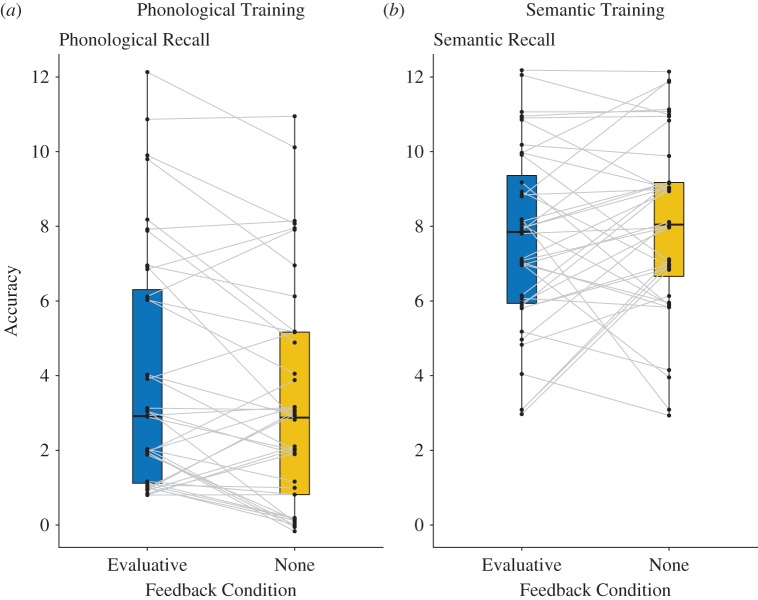
Individual differences in cued-recall accuracy in the phonological and semantic training conditions. Specifically, panel (*a*) shows the effect of feedback on cued recall of phonology for individuals in the phonological training condition. Panel (*b*) shows the effect of feedback on cued recall of semantics for individuals in the semantic training condition. The influence of evaluative feedback is significant for (*a*), but not (*b*). The black dots represent the performance of individual participants; the grey lines depict the change in performance across feedback conditions for each individual. Points are vertically jittered in order to show data from all the participants. The edges of the boxes on the box plots depict the first and third quartile; the whiskers show the upper and lower extremes. The median is shown within the box.

As stated above, given that phonological learning in the semantic training condition was limited (and no feedback was received for phonology in the semantic training condition), we decided to examine whether the effect of feedback on phonological recall held independently for phonological recall in the phonological training condition. Items where evaluative feedback was given (M = 4.0, s.d. = 3.3) were recalled more accurately than those where no feedback was given (M = 3.3, s.d. = 3.0), *t*_39_ = 2.82, *p* = 0.008, *d* = 0.45 (figure 3*a*). This is significant even at a Bonferroni-corrected threshold of *p* < 0.0125, which is *p* < 0.05 corrected for four comparisons. By contrast, for semantic cued recall in the semantic training condition, there was no evidence that feedback influenced recall, *t*_39_ = 1.1, *p* = 0.27, *d* = 0.18 (figure 3*b*).

We also assessed whether phonological cued recall was influenced by feedback in the semantic training condition. Here, we found participants were at floor both with (M = 0.4, s.d. = 0.8) and without evaluative feedback (M = 0.4, s.d. = 0.7), with no significant difference between feedback conditions, *t*_39_ = 0.35, *p* = 0.73, *d* = 0.06. This suggests that, as we might expect, the effect of feedback on cued recall of phonology is driven by items in the phonological training condition. For semantic cued recall in the phonological training condition, again, there was no evidence that feedback influenced recall. Items that received feedback (M = 1.1, s.d. = 1.4) were recalled as accurately as those that did not (M = 1.4, s.d. = 1.8), *t*_39_ = 1.15, *p* = 0.26, *d* = 0.18.

In responding to one of the reviewers of our Stage 1 manuscript, we proposed to examine whether the effects of feedback were due to the presence of greater errors in the phonological training condition relative to the semantic training condition. In electronic supplementary material, appendix S4, we present analyses we originally proposed to address this question, that is, comparing learning of the easier two-syllable forms to the harder abstract facts. However, one of the issues with this analysis was the potential lack of sensitivity, as each analysis necessarily entailed losing half our dataset. We therefore decided to probe whether there were any interactions with phonological and semantic difficulty within our dataset.

#### 3.5.2. Interaction of feedback with phonological difficulty

To assess whether feedback had differential effects when learning two-syllable or three-syllable words, we conducted an ANOVA assessing the influence of feedback and syllable condition, only focusing on participants in the phonological training condition completing phonological recall. The interaction between syllable condition and feedback condition was not significant, *F*_1,39_ = 0.1, *p* = 0.76, *η*_G_^2^ = 0.0002. As previously reported, we noted a significant main effect of feedback condition, *F*_1,39_ = 7.9, *p* = 0.008, *η*_G_^2^ = 0.01, as well as a main effect of syllable condition, *F*_1,39_ = 49.0, *p* < 0.001, *η*_G_^2^ = 0.09.

#### 3.5.3. Interaction of feedback with semantic difficulty

For comparison with the previous analysis, we assessed whether feedback had differential effects when learning observable or abstract facts. We conducted an ANOVA assessing the influence of feedback and observability for participants in the semantic training condition completing semantic recall. Again, we found that the interaction between semantic condition and feedback condition was not significant, *F*_1,39_ = 0.4, *p* = 0.52, *η*_G_^2^ = 0.002. As previously highlighted, feedback did not influence performance on semantic recall, *F*_1,39_ = 1.2, *p* = 0.27, *η*_G_^2^ = 0.004. A main effect of observability was observed, *F*_1,39_ = 81.2, *p* < 0.001, *η*_G_^2^ = 0.27, indicating that observable facts were recalled more accurately than the abstract ones at final test.

#### 3.5.4. Training data

**Figure 4.**
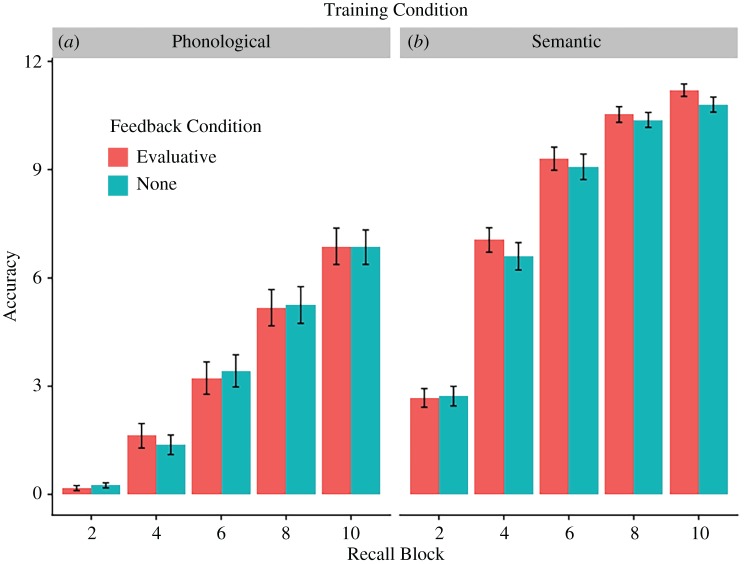
Mean recall accuracy in the two training conditions, (*a*) phonological and (*b*) semantic. Red bars show performance for items that received evaluative feedback, and blue bars show performance for the items when no feedback was received. Performance is grouped by block, only blocks 2, 4, 6, 8 and 10 are shown, as blocks 1, 3, 5, 7, 9 and 11 are blocks where participants had to repeat the phonological form or semantic fact. Error bars denote ±1 standard error of the mean.

We assessed whether similar patterns of learning were observed for the semantic and phonological training conditions using a 2 (training condition: phonological/semantic) × 5 (cued-recall block: 2/4/6/8/10) ANOVA. We observed a main effect of training condition, *F*_1,78_ = 153.9, *p* < 0.001, a main effect of block, *F*_1,78_ = 847.4, *p* < 0.001, which was qualified by an interaction between training condition and block, *F*_1,78_ = 6.3, *p* = 0.014. Participants in the semantic training condition learned more facts (M = 8.0, s.d. = 3.5) than participants in the phonological condition, who learned fewer phonological forms (M = 3.4, s.d. = 3.5). As expected, performance improved over blocks (Block 2: M = 1.5, s.d. = 1.7; Block 4: M= 4.2, s.d. = 3.4, Block 6: M = 6.3, s.d. = 3.8; Block 8: M = 7.8, s.d. = 3.6; Block 10: M = 8.9, s.d. = 3.1). The interaction denoted different slopes of learning across the two training conditions; figure 4. Feedback did not significantly moderate performance during training, *F*_1,78_ = 0.8, *p* = 0.371, and no interactions of feedback with block, *F*_1,78_ = 0.2, *p* = 0.691, training condition, *F*_1,78_ = 1.0, *p* = 0.309, or training condition and block, *F*_1,78_ = 0.4, *p* = 0.544, were noted.

However, it is important to bear in mind that performance during training may not be an adequate measure of participants' learning, and that a test at the end of the learning block may have yielded different results.

#### 3.5.5. Error analysis from the last block of training

**Figure 5.**
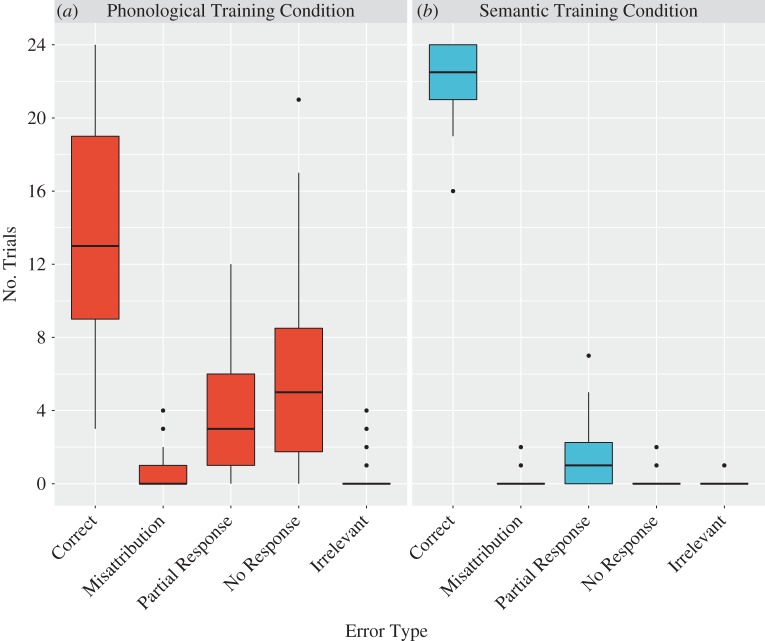
Box plot showing the distribution of different error types in the last block of training. Red boxes depict performance in the phonological training condition and the blue boxes show performance in the semantic training condition. See the legend to figure 2 for details.

Errors made by a participant could also shed light on the aspect of learning they found challenging, and this might differ across the two training conditions. For example, we predicted that a lack of response was likely to be the most common error in the phonological training condition, whereas a closely related response was most likely in the semantic training condition. We therefore assessed the frequency of different types of errors in the last cued-recall block of the two training conditions. We coded these in the last block to understand how participants were encoding semantic/phonological information. We coded errors as one of four types, (1) misattribution of referent (wrong phonological form or wrong fact associated with target), (2) partially correct response (in the phonological condition, saying ‘mamogan’ instead of mamogus, and in the semantic training condition, saying ‘hupip has multiple tendrils', instead of ‘hupip has several tendrils’), (3) no response, and (4) irrelevant response. Correct trials were coded as correct. In the last block of the phonological training condition, participants performed accurately in 13.7 (s.d. = 5.9) of 24 trials. When they were inaccurate, they misattributed a phonological form to a referent in 0.5 (s.d. = 0.9) of 24 trials, gave a partially correct response in 3.8 (s.d. = 3.1) of 24 trials, gave no response at all in 5.7 of 24 trials (s.d. = 5.3) and gave an irrelevant response in 0.4 of 24 trials (s.d. = 0.9) (figure 5). In the last block of the semantic training condition, participants performed accurately in 22 (s.d. = 2.1) of the 24 trials. Of the few errors made, most indicated gist recall of the fact but not the specific wording: 1.6 (s.d. = 1.7) partially correct. Very small numbers of errors were due to misattribution of facts to referents (0.1, s.d. = 0.4), no responses (0.3, s.d. = 0.6) or irrelevant responses (0, s.d. = 0.2) (figure 5).

## 4. Discussion

In this study, we assessed whether evaluative (right/wrong) feedback would influence long-term word learning, and whether such feedback would have differential effects on the learning of phonological forms and semantic facts. Our results indicate that evaluative feedback can improve retention of phonological forms, but such feedback does not appear to be of benefit for semantic learning. This suggests that it may be more beneficial to give evaluative feedback when targeting phonological learning, rather than when targeting semantic learning. Although we have explored evaluative feedback here, it may be the case that evaluative feedback combined with providing the right answer could yield even greater benefits during phonological learning. A role for external feedback in shaping phonological learning is potentially exciting from the perspective of designing more effective training programs. We also find that directing attention to semantic or phonological cues during training is remarkably effective in achieving learning of semantics or phonology, respectively, but that there is relatively little transfer to the other domain. Again, this finding suggests that, for optimal learning, it is important to target both semantic and phonological aspects of word learning during training.

The conclusions about feedback are worth commenting on in the context of our pre-registered analyses. Strictly speaking, we expected to find a significant interaction between feedback and training condition, or a three-way interaction between feedback, cued-recall condition and training condition. The interaction between feedback and training condition, and the three-way interaction were not significant. However, we did not find this to be convincing evidence to reject the hypothesis that feedback affects learning for two reasons. One, we observed a significant interaction between feedback and cued-recall condition, which we had anticipated. Two, we observed floor effects for phonological learning in the semantic training condition, and vice versa. Given these floor effects, we thought it was valid to assess separately the effect of feedback on phonological learning in the phonological training condition, and the effect of feedback on semantic learning in the semantic training condition. These analyses revealed a small but significant effect of feedback in the phonological training condition, and no effect of feedback in the semantic training condition.

Splitting up the analysis by the two conditions was also supported by the overall difference in learning across the two conditions. Average performance was quite poor in the phonological training condition. At the final test, participants remembered an average of 30.2% of the 24 words they encountered. This was in contrast to the semantic condition, where participants remembered an average of 65.3% of the 24 facts they encountered at the final test. We had attempted to mitigate differences across the two training conditions by conducting pilot testing with the stimuli used here (see electronic supplementary material, appendix S3). However, phonological learning appears to be characterized by wide individual differences, even in healthy adults primarily drawn from the undergraduate student body at the University of Oxford. Consequently, our pilot data were not representative of the full sample, and at test we found that phonological forms were more difficult to encode than semantic facts. In future work, improving group performance on phonological form learning, and therefore avoiding the possibility of floor effects, will be an important step. To achieve this, one thing we have considered changing is the number of stimuli to be learnt. On the one hand, to reliably estimate performance for an individual, averaging across as many stimuli as possible is ideal. Here, our average scores in each condition reflect performance across 12 stimuli. On the other hand, including more stimuli also increases the burden on the learner. One way to mitigate this might be to use between-subject designs, where participants in one group are exposed to one condition, and those in a different group are exposed to the other. However, this does mean collecting data from more participants to achieve the same power. Furthermore, the individual variation observed in this study suggests it may be difficult to balance the learning ability of the two groups.

Seen in the light of overall poor performance for phonological learning, the effect of evaluative feedback on learning phonological forms is promising. In previous work, we have found it quite difficult to affect phonological learning outcomes by changing the way information is presented during training [40]. Although the effect of evaluative feedback is small, it is consistent across individuals, particularly those who are not at floor (figure 3*a*). This does suggest there is a role for explicit, direct feedback in word learning, something most researchers have argued is a relatively implicit process. This role has been dismissed in other studies [19]; however, unlike our study, previous experiments did not allow for opportunities for re-exposure during training. The dynamic nature of our training may have allowed participants to use correct/incorrect feedback to reinforce responses they were not sure about, and use the re-exposure opportunities to learn forms they incorrectly remembered. In contrast to learning phonological forms, we found that evaluative feedback had little effect on learning semantic facts. This is inconsistent with some other findings that suggest feedback does influence memory for semantic facts [29]. However, this could again be explained by differences in our paradigm, which offered multiple learning opportunities rather than a single instance of feedback at the end of learning. It is possible that feedback has an influence on semantics when only a single testing opportunity is available, but this influence might be reduced when multiple testing opportunities are available. A similar point was made by Hays *et al.* [17], who showed that when feedback was given in lieu of testing opportunities, feedback appeared to have little effect on learning.

Why was evaluative feedback effective when participants were learning phonology, but not when they were learning semantics? One reason might be that we were more sensitive to performance differences in the phonological training condition, as participants were nearly at ceiling towards the end of semantic training. However, although participants learned fewer phonological forms than semantic facts, semantic recall a week after semantic training was not close to ceiling. Instead, the mean accuracy was approximately 65%. Therefore, a difference in sensitivity is unlikely to account for the differences observed in the two conditions. Another explanation derives from the fact that feedback appears to be beneficial when learning is difficult [41]. Indeed, participants have been shown to benefit from the errors they make during learning [15]. This is probably because the discrepancy between the error and subsequent feedback captures the participants' attention, and enhances encoding of the correct answer. As we did note that phonological learning was more difficult than semantic learning, could differences in difficulty explain the pattern of data we observe? In exploratory analyses, we probed whether feedback had different benefits for learning easier/harder phonological forms; or easier/harder semantic facts. For learning two- and three-syllable words, we did not find an interaction with syllable condition. The interaction between feedback and semantic condition was also non-significant. This suggests that difficulty may be an incomplete or insufficient explanation for the differences observed across the two conditions.

The errors made by a participant could also shed light on the aspect of learning they struggle with. For instance, an error could be driven by participants not attaching a fact or form to the correct visual referent (misattribution). Alternatively, participants might partially produce the fact or form, suggesting some access to the representation that can be corrected. Finally, they may not produce any response at all, or come up with an irrelevant answer (for example, ‘The plant's name is Tom’). The frequency of these kinds of errors is likely to differ across the semantic and phonological learning conditions. To address this, we examined the type of errors participants made in the two training conditions, by examining learning in the final block of training. We chose to examine the final block of learning, rather than the final test, as this measure was more indicative of performance during encoding. Encouragingly, very few errors were due to misattribution, indicating that participants were not simply mis-remembering the association between a referent and a response. We found that errors during the last block of phonological training were most likely to be a lack of any response at all, closely followed by those where participants gave partially correct responses. The most frequent error type in the last block of semantic training was giving a partially correct response. However, partially correct responses do represent different kinds of attempts in the semantic and phonological conditions. When receiving feedback that a sequence of sounds is incorrect, for example, saying ‘mamogan’ instead of ‘mamogus’, participants may have been motivated to pay closer attention to the sequence in the following repetition trial. However, when receiving feedback that a set of words was incorrect, despite capturing the semantic gist (for example, saying ‘has orange leaves’ instead of ‘has orange tendrils’), participants might be unable to appreciate why their representation needed updating, causing them not to benefit from feedback.

Indeed, the reason that feedback might have differential effects during semantic and phonological learning is that these two forms of learning engage differential processing mechanisms during encoding and consolidation. For phonological learning, participants have to compute a new sensorimotor transformation, of a novel sequence of sounds, and produce the corresponding motor pattern with their oral articulators [42,43]. They have to store this sensorimotor sequence in memory, and then recall it in association with a visual referent. Our previous work suggests that retrieval, reproduction or auditory exposure to phonological forms results in equivalent long-term phonological learning [40]. Receiving external feedback after a production attempt might strengthen a nascent phonological representation, and equally weaken an incorrect representation. In contrast, for semantic learning, participants believe they have to retrieve a gist, rather than the correct combination of words (although they are instructed to retrieve the precise definition). Here, evaluative right/wrong feedback may do very little to shape access to the precise semantic representation. Meta-cognition, or one's ability to judge their own learning, may also play a role in learning. For example, differences in meta-comprehension are linked to learning from text passages. Learners who were better able to judge their own learning accuracy improved after a period of restudy, while those with poor judgement accuracy did not really improve [44]. Meta-cognition is likely to differ across the two domains we tested here. Participants are likely to be quite good at judging whether their semantic responses are right or wrong, perhaps making the evaluative feedback they receive redundant. On the other hand, when learning phonological forms, participants may not feel confident about their productions, especially when they produce a response close to the target but not quite the right answer. Receiving evaluative feedback could cue them to pay closer attention to a phonological form if they were incorrect, and strengthen their representation of the form if they were correct. Evaluative right/wrong feedback may therefore allow participants to improve their meta-cognition of phonological learning.

The purpose of using simple evaluative feedback in this study was to assess whether receiving an error signal on performance could boost learning of phonology and semantics. However, previous research indicated that other forms of feedback, such as receiving the correct answer, would lead to better retention outcomes [45]. It is also more naturalistic to receive feedback such as ‘Correct, it's the bazo’, or ‘No, that's a hupip, not a hutip’. Future studies are needed to explore what additional benefit such re-exposure might have, and whether evaluative feedback confers a learning boost beyond re-exposure to the correct form alone. It is possible that feedback that includes the correct answer, or provides more semantic context, might also be more effective when one engages in semantic learning. In future studies, it would also be worth exploring how changes in the value of feedback that participants receive influence learning. For example, feedback that is more rewarding might be associated with greater learning relative to evaluative right/wrong feedback.

It is also important to note that we only observed effects of feedback on the final test, which occurred one week after learning. Somewhat surprisingly, we did not observe an effect of feedback in the training trials. It is premature to comment on how feedback might differentially affect different points of the learning trajectory, as we did not intend to use the training data to assess the benefits of feedback on learning. Indeed, we have not conducted direct comparisons between the final test and training blocks. In addition, participants did receive an additional exposure to the phonological form/semantic fact even after the last cued-recall block, which may have influenced the pattern of retention we observe. However, this suggests the effects of feedback on vocabulary learning may only be evident in the longer term, when participants start to forget what they have previously learned. This issue needs to be evaluated in future studies.

Beyond the effects of feedback, we found that directing attention to semantic facts did not have a facilitatory effect on phonological learning, a result that might appear to be at odds with other findings in the literature [30,33]. However, previous studies have typically contrasted phonology presented in rich semantic contexts, such as a story, to completely sparse presentations of phonology. An advantage of our design was that the same material was presented in both the phonological and semantic training conditions, with attention being directed to either semantics or phonology. Our data suggest that designs used in previous studies may actually boost overall motivation or attention to phonology, rather than provide a boost to memory by giving information about the meaning of the referent. However, our data do not indicate that providing semantic context is of no benefit at all during learning. One issue with our design is that participants in the semantic training condition were never given an opportunity to practice phonological recall, and vice versa. A follow-up study using the same stimuli, where participants are given some opportunities to learn phonology/semantics during training, could indicate whether a small amount of attentional redirection could shape phonological or semantic learning differently. A related issue is that not all learners will benefit equally from semantic information. In a recent study where children learned word meanings through incidental exposure in print, traditional pen-and-paper-based tests showed no difference between words encountered in more diverse semantic contexts to those in less diverse contexts. However, these results were mediated by comprehension skill, with better comprehenders able to benefit from a reduction in reading times for words encountered in more diverse contexts. Poor comprehenders did not show this reduction [46]. In future work, it is necessary to evaluate how individual differences in IQ, language competence and verbal memory might influence performance. Our previous work [40] suggested that verbal memory was strongly tied to cued recall of phonology, but so far we have not explored factors that might shape lexical learning (see [47] for a discussion).

It would be tempting to apply this conclusion to other populations like children or those with word learning difficulties, but further research is necessary to evaluate whether our findings would hold in populations beyond healthy adults. For example, children do not always benefit from the same learning conditions as adults [48,49]. Furthermore, if the goal is to use these conditions to train those with language disorders, it will be necessary to evaluate the effectiveness of these learning conditions with these groups. For example, when learning to understand spatial prepositions, Hsu and Bishop [50] found that children with developmental language disorders benefitted from multiple exposures to the same form. This is different from what might be expected from typically developing children, for whom variability appears to be key to learning structure.

## 5. Summary and conclusion

Our data indicate that evaluative feedback confers a small benefit to long-term phonological learning when dynamic learning paradigms are employed. In other words, receiving evaluative or right/wrong feedback on overt pronunciation attempts appears to confer a slight advantage when learning new words. However, the same feedback advantage is not observed for learning semantic information linked to the target. We have hypothesized that other kinds of feedback may be more suitable in these conditions, such as receiving the correct answer or related semantic information. Our findings also suggest that actively directing attention towards the phonological form and semantic facts during training would be of benefit during word learning training, rather than just providing phonological forms within a semantic context.

## Supplementary Material

Stage2_Appendix

## Data accessibility

Our data have been deposited on the Open Science Framework (OSF), and are accessible via https://osf.io/bdfqy/ (doi:10.17605/osf.io/bdfqy) [34]. Code to run the training program, as well as to analyse the data are also available on the Open Science Framework via the same link.
