# Supplementary material for: The influence of evaluative right/wrong feedback on phonological and semantic processes in word learning
Source: R Soc Open Sci. 2018 Sep 5;5(9):171496. doi: 10.1098/rsos.171496 (PMC6170543; doi:10.1098/rsos.171496)
Supplement: Stage2_Appendix [file rsos171496supp1.docx]

**Appendix 1: Results of pilot testing**

*Method*

Participants had to learn pseudowords and semantic facts about 24 novel visual stimuli they had not encountered before, drawn from 2 matched lists comprising 12 items each. Eight participants (between the ages of 18-40 years and native English speakers) gave informed consent and completed pilot testing (four were assigned to the semantic training condition and four to the phonological training conditions). In the semantic condition, participants repeated or recalled the semantic fact they heard, whereas in the phonological condition, they were asked to repeat or recall the pseudoword. Evaluative right/wrong was given for items from one of the lists, the other items did not receive any feedback. Across participants, list order and feedback assigned to each list were fully counterbalanced. Participants were exposed to eleven blocks of training, alternating between “Repeat” and “Retrieve” blocks. However, experimenters had the option to stop after block 7 and 9 if the participants were fatigued or were at ceiling. Participants in the phonological training condition completed all 11 blocks, however, 2 participants in the semantic training condition completed only 9 blocks (they did not do the last retrieve and repeat block). After training, participants were immediately tested on their recall of both phonological forms and semantic facts, regardless of the training condition they were exposed to. The order in which cued recall of phonology and semantics was tested was also counterbalanced across participants.

*Results and discussion*

During the course of training, participants in the phonological training condition showed learning of pseudowords, and those in the semantic training condition showed learning of semantic facts (Figure 1). Participants received one further reproduction block after block 10, before they attempted the final tests. Note that two participants in the semantic condition did not complete block 10 and 11.


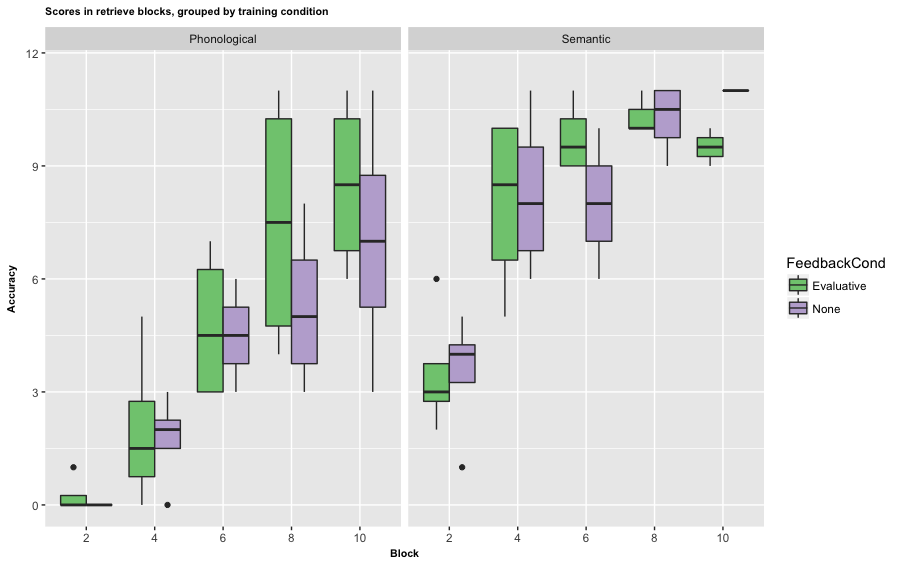


Figure 1. Boxplot showing accuracy in the retrieve blocks during training, grouped by training condition and whether feedback was provided. The black lines show median scores in each block, the upper and lower bounds of the box denote the 75^th^ and 25^th^ quartile respectively and the whiskers denote the maximum/minimum in the condition excluding outliers.

At the end of training, participants completed tests assessing their cued recall of semantics and phonology. This data conformed to the pattern we predicted, and also suggested that there were no ceiling effects in semantic scores. Due to the small sample size, we did not run statistical tests to assess differences.


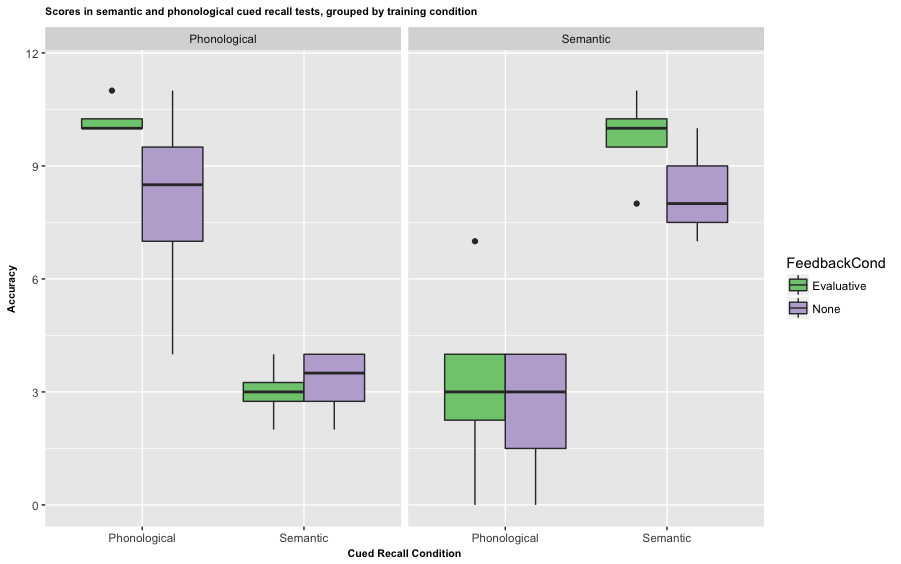


Figure 2 depicts performance in the final recall tests of phonology and semantics, grouped by training condition and whether the participants received evaluative feedback or not.

Although we initially planned to include only 6 blocks during training (with blocks 2, 4 & 6 being retrieve blocks), we find that this would lead to substantial difference in accuracy between the phonological and semantic training conditions (38.54% and 78.13% respectively). Indeed, we conducted the piloting we originally proposed in the revision with 2 participants (4 interleaved repeat and recall blocks) only to find that participants were nearly at floor, which led us to use this longer training program. However, by the 10^th^ block, we find that participants reach a relatively similar and high level of accuracy across the two conditions (79.17% and 87.5% for the phonological and semantic training condition respectively). We therefore propose using the 11 block regime for training, with blocks 2,4,6,8, and 10 being retrieve blocks. This would allow participants 6 exposures to learn the words/ facts during training.

After 11 blocks of training, scores in the final recall tests remain high for the condition participants are trained for (76.04% and 79.17% for phonological and semantic recall in the phonological and semantic training condition respectively). However, we believe that we are unlikely to encounter ceiling effects as our final tests will be conducted one week after training. Testing participants one week later is likely to be associated with a fall of ~20% in accuracy (for instance, this pattern is seen in Krishnan, Watkins, & Bishop, 2017, *BMC Psychology*).

*** Pilot data and analysis scripts are available at* ***https://osf.io/bdfqy/***
